# Comparison of Different Cooling Schemes for AlGaN/GaN High-Electron Mobility Transistors

**DOI:** 10.3390/mi15010033

**Published:** 2023-12-23

**Authors:** Yunqian Song, Chuan Chen, Qidong Wang, Jianyu Feng, Rong Fu, Xiaobin Zhang, Liqiang Cao

**Affiliations:** 1 Institute of Microelectronics of Chinese Academy of Sciences, Beijing 100029, China; songyunqian@ime.ac.cn (Y.S.); wangqidong@ime.ac.cn (Q.W.); fengjianyu@ime.ac.cn (J.F.); furong@ime.ac.cn (R.F.); zhangxiaobin@ime.ac.cn (X.Z.); 2 University of Chinese Academy of Sciences, Beijing 100049, China; 3 State Key Lab of Fabrication Technologies for Integrated Circuits, Institute of Microelectronics, Chinese Academy of Sciences, Beijing 100029, China; 4 Leihua Electronic Technology Research Institute AVIC, Wuxi 214063, China

**Keywords:** AlGaN/GaN HEMTs, remote cooling, near-chip cooling, chip-embedded cooling, hotspot

## Abstract

Cooling is important for AlGaN/GaN high-electron mobility transistors (HEMTs) performance. In this paper, the advantages and disadvantages of the cooling performance of three cooling schemes: remote cooling (R-cool), near-chip cooling (NC-cool), and chip-embedded cooling (CE-cool) are compared. The influences of distinct geometric parameters and operating conditions on thermal resistance are investigated. The results show that the thermal resistances of NC-cool and CE-cool are almost the same as each other. Decreasing microchannel base thickness (*h_b_*) significantly increases the thermal resistance of CE-cool, and when its thickness is less than a critical value, NC-cool exhibits superior cooling performance than CE-cool. The critical thickness increases when decreasing the heat source pitch (*P_h_*) and the convective heat transfer coefficient (*h_conv_*) or increasing the thermal conductivity of the substrate (*λ_sub_*). Moreover, increasing *P_h_* or *λ_sub_* significantly improves the thermal resistance of three cooling schemes. Increasing *h_conv_* significantly decreases the thermal resistances of NC-cool and CE-cool while hardly affecting the thermal resistance of R-cool. The influence of the boundary thermal resistance (*TBR*) on the thermal resistance significantly increases at higher *λ_sub_* and larger *h_conv_*.

## 1. Introduction

AlGaN/GaN HEMTs have been widely used in 5G communication, radar, and electronic warfare due to their unique advantages including low parasitic inductance, high operating frequency, and high power density. The heat flux of GaN devices in the future will reach 1000 W/cm^2^, and the local heat flux may reach as high as 30 kW/cm^2^ [1,2,3], while the junction temperature of the devices should be kept below 200 °C [4]. In the lab, AlGaN/GaN HEMTs can generate power of more than 40 W/mm [5]. However, the actual power density is currently only 4–5 W/mm [6,7] due to the limitation in cooling capacity. Particularly, the hotspot generated by the accumulation of heat in the near-junction region leads to serious degradation, for example, in gain, output power, and power-added efficiency [8,9], limiting the performance of high-power output. Therefore, cooling technology has become the key to further enhancing the performance of AlGaN/GaN HEMT devices.

The key to the thermal design and optimization of AlGaN/GaN HEMT devices is to analyze the thermal characteristics of AlGaN/GaN HEMT devices and understand the influences of geometric parameters on the junction temperature and thermal resistance of the devices. Some researchers have studied the dependence of the junction temperature and thermal resistance of AlGaN/GaN HEMT devices on the geometrical parameters of the device structure using finite element simulation. Guo et al. [10] used the finite element method to conduct a three-dimensional thermal simulation analysis on the heat dissipation of Diamond substrate AlGaN/GaN HEMTs. The results showed that the thickness of the epitaxial layer and boundary thermal resistance has a certain influence on the magnitude of the junction temperature, especially the boundary thermal resistance, of which the effect is up to 19 K/10 m^2^·K·GW^−1^. Gerrer et al. [11] used finite element simulation to analyze the influences of geometric parameters on the thermal characteristics of Diamond substrate AlGaN/GaN HEMTs. The results showed that GaN-on-Diamond with a 13 μm gate pitch provides three times the surface power density compared with GaN-on-SiC with a 40 μm gate pitch. Kim et al. [12] analyzed the influences of a GaN layer and Diamond substrate thickness, GaN/Diamond thermal boundary resistance, gate pitch, and width on the junction temperature of GaN-on-Diamond devices by constructing a simplified three-dimensional near-junction heat transport (NJTT) model of heterogeneous integration of high-heat-flux GaN devices. The results showed that when the boundary thermal resistance is 30 and 6.5 m^2^·K·GW^−1^, the optimal values of GaN layer thickness to reach the minimum junction temperature and thermal resistance of the devices are 5.9 and 3.5 μm, respectively. Previous work focused on studying the influences of the geometrical parameters of the device structure on the thermal characteristics of GaN HEMTs, including gate pitch and gate width, epitaxial layer thickness, substrate material, and boundary thermal resistance of the heterogeneous interface. However, less attention has been paid to the influences of the interaction between the convective heat transfer coefficient, microchannel base thickness, and some device geometric parameters on the thermal characteristics of AlGaN/GaN HEMT devices in integrated cooling applications.

**Figure 1:**
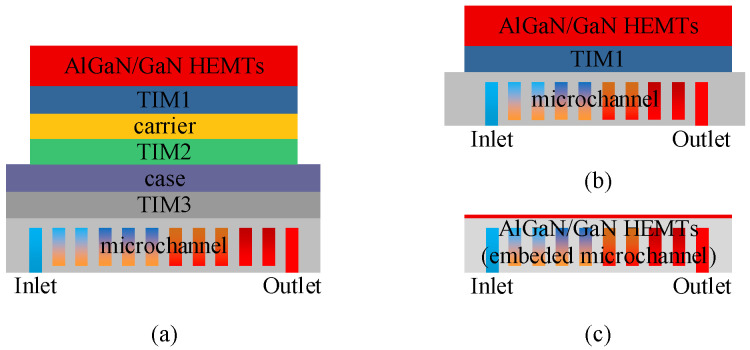
Three cooling schemes for AlGaN/GaN HEMTs: (**a**) R-cool, (**b**) NC-cool, and (**c**) CE-cool.

Currently, there are three main cooling schemes for AlGaN/GaN HEMT device integration, as shown in Figure 1. The first scheme is traditional remote cooling (R-cool) (Figure 1a), where the devices are integrated onto the carrier via TIM1, then onto the case via TIM2, and further integrated onto the microchannel cold plate via TIM3 for heat dissipation. This scheme has high thermal resistance. The second scheme is near-chip cooling (NC-cool) (Figure 1b), where the devices are integrated onto the microchannel cold plate via TIM1. Compared with R-cool, NC-cool enhances the thermal dissipation performance by shortening the heat transfer path. In recent years, the use of a 2.5D microfluidic silicon interposer to achieve NC-cool of GaN HEMTs has attracted great attention. Lian et al. [13] proposed a 2.5D silicon interposer integration method with microchannel direct cooling for a high-power GaN HEMT device. The experimental results showed that the maximum surface temperature of the device is 93.8 °C when the heat flux in the gate finger region is 32 kw/cm^2^. Miao et al. [14] demonstrated a microfluidic silicon interposer based on microjet cooling for the thermal management of GaN device integration, with a chip-level heat flux greater than 500 W/cm^2^ and a hotspot heat flux greater than 30 kW/cm^2^. Ditri et al. [15,16] explored a microfluidic cooling method that combines a pin-fin structure and a microfluidic impingement microjet to achieve the cooling of GaN devices with a chip-level heat flux greater than 1 kW/cm^2^ and a hotspot heat flux greater than 40 kW/cm^2^. The third option is chip-embedded cooling (CE-cool) (Figure 1c), where microchannels are etched directly onto the backside of the device substrate. Compared with NC-cool, the CE-cool scheme eliminates TIM and further shortens the heat transfer path. Since the Defense Advanced Research Projects Agency launched the Intra/Interchip Enhanced Cooling (ICEcool) Program, the CE-cool scheme has become another research hotspot for the thermal management of high-power GaN HEMTs. Jung et al. [17,18,19] designed an embedded microchannel cooling structure with a three-dimensional silicon manifold and a cooling area of 5 × 5 mm^2^. When the heat flux is 250–300 W/cm^2^, the maximum device temperature is 80–90 °C. Erp et al. [20] demonstrated a chip-embedded microfluidic cooling method that can efficiently cool the heat flux up to 1.7 kW/cm^2^, while the pumping power is only 0.57 W/cm^2^.

In the process of heat transfer from the hotspot to the coolant, there are differences in vertical conduction and transverse diffusion capabilities under different cooling schemes and different structures. The closer the coolant is to the heat source does not inevitably lead to a smaller thermal resistance and thus, to better thermal characteristics. In terms of comparison of different cooling schemes, Chen et al. [21] numerically compared the active and passive cooling of AlGaN/GaN HEMTs, and the simulation results showed that a Diamond substrate proved to be superior in reducing the junction temperature in passive cooling methods using a high thermal conductivity substrate. However, single-phase liquid cooling with microchannels integrated into a SiC substrate could dissipate higher power densities than on a 200 μm thick Diamond substrate. Erp et al. [22] proposed a microchannel cooling structure of GaN devices with a three-dimensional manifold. The experimental results showed that the structure can achieve a power dissipation of 600 W while keeping the junction temperature rise below 60 °C. Compared with traditional cooling, the power density is increased by 10 times, potentially up to 30 kW/cm^3^. Ye et al. [23] investigated the thermal characteristics of GaN HEMTs with embedded microfluidic cooling. The experimental results showed that the heat flux at the gate finger hotspot can reach 6349.2 W/mm^2^, which is more than 10 times that of traditional remote cooling.

However, so far, few research studies have investigated the thermal characteristics analysis of the three cooling schemes, especially the comparative analysis of NC-cool and CE-cool. This study aims to fill this gap. In this paper, the thermal characteristics of three cooling schemes for AlGaN/GaN HEMTs, namely, R-cool, NC-cool, and CE-cool, are compared and analyzed. Firstly, a three-dimensional finite element steady-state thermal analysis model is established, and then the thermal characteristics of three cooling schemes are compared. Finally, the influence rules and mechanisms of some geometric parameters and working conditions on thermal resistance are analyzed, including heat source pitch, the thermal conductivity of the substrate, convective heat transfer coefficients, the boundary thermal resistance of heterogeneous interface, microchannel base thickness, and their interaction factors. This research results provide a valuable reference for the reasonable selection and optimal design of cooling schemes for AlGaN/GaN HEMTs.

## 2. Modeling and Simulation

### 2.1. Model and Materials

**Figure 2:**
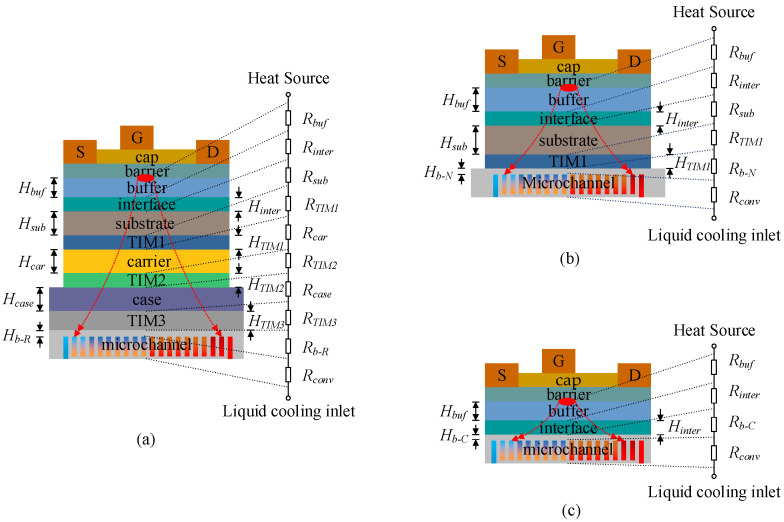
Schematic cross-sectional diagram and thermal resistance modeling of three cooling schemes for AlGaN/GaN HEMTs: (**a**) the R-cool scheme, (**b**) the NC-cool scheme, and (**c**) the CE-cool scheme.

Figure 2 shows a schematic cross-sectional diagram [24,25,26,27] and the thermal resistance model of three cooling schemes for AlGaN/GaN HEMTs, which represents the heat transfer path from the heat source to the coolant. *R_buf_*, *R_inter_*, *R_sub_*, *R_TIM1_*, *R_car_*, *R_TIM2_*, *R_case_*, *R_TIM3_*, *R_b_*, and *R_conv_*, respectively, represent the conduction thermal resistance of the buffer layer, interface layer, substrate layer, TIM1 layer, carrier layer, TIM2 layer, case layer, TIM3 layer and microchannel base layer and the convection thermal resistance of microchannel.

To compare the thermal characteristics of the different cooling schemes, a three-dimensional finite element analysis was conducted to study the steady-state heat transfer with Ansys Workbench 2022 R1 software. In this work, the same structure of AlGaN/HEMTs is used for the three cooling schemes, including an active layer such as source and drain, the AlGaN barrier layer, the GaN buffer layer, and the substrate layer. The type of the device structure is transverse AlGaN/GaN HEMTs, and the device is a heterojunction device. An AlGaN/GaN heterojunction is formed by growing an AlGaN layer on a GaN layer with vapor deposition or molecular beam epitaxy. The following assumptions are made to simplify the computation:

(1) The heat source of the AlGaN/GaN HEMTs device is located in the submicron region below the gate finger toward the drain side [12,28,29], which has the same dimensions as the gate. To simplify the model, the gate, source, and drain are omitted.
(2) All heat is conducted downward, and the thicknesses of the upper materials are all in the order of nanometers, which has a minimal influence on the simulation [30,31,32]. In this model, the AlGaN barrier layer and the GaN cap layer are ignored.
(3) The *TBR* of the heterogeneous interface between GaN and SiC significantly affects the overall thermal resistance of GaN devices [33], which cannot be ignored. In order to generate a smoother mesh and save computational resources, it is equivalent to a thicker anisotropic material in this model, and the thermal conductivity is set to be *λ_inter_* in the direction of thermal conduction and 0 in the other directions. *λ_inter_* is calculated as follows,

(1) $$\lambda_{inter}=\frac{H_{inter}}{TBR}$$

where *H_inter_* is the equivalent thickness and *TBR* is the boundary thermal resistance of the heterogeneous interface.

(4) Other interface thermal resistances are ignored.
(5) The change in thermal conductivity of the material with temperature and the influence of thermal radiation are not considered.

**Figure 3:**
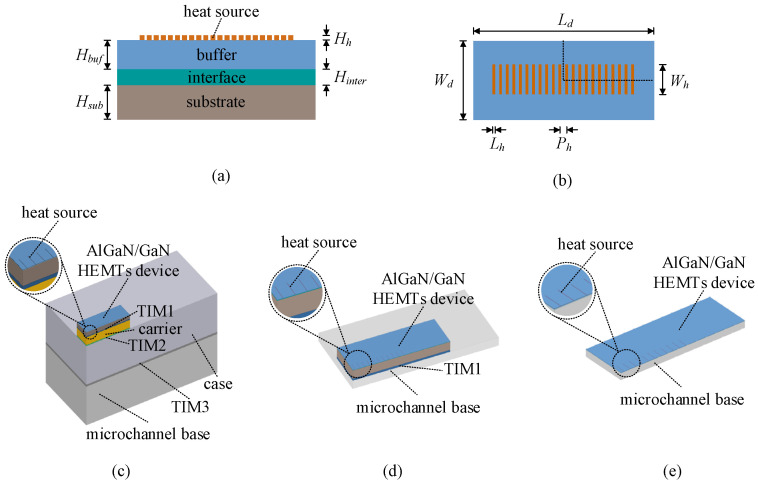
Three-dimensional simulation models. (**a**) Schematic cross-sectional diagram of the AlGaN/GaN HEMT device. (**b**) Top view of the AlGaN/GaN HEMT device. (**c**) The R-cool scheme. (**d**) The NC-cool scheme. (**e**) The CE-cool scheme.

According to the above assumptions, the device in the model consists of a heat source layer, a GaN buffer layer, an AlN interface layer, and a SiC substrate layer from top to bottom, and the thickness is *H_h_*, *H_buf_*, *H_inter_*, and *H_sub_*, respectively, as shown in Figure 3a. Figure 3b shows the top view, and the heating region in the model contains 22 heat sources [34]. The length of the heat source is *L_h_*, the width is *W_h_*, the height is *H_h_*, and the heat source pitch is *P_h_*. Considering the symmetry of the structure, a quarter model is chosen to reduce the computational amount, and the adiabatic boundary conditions are imposed on the two symmetry surfaces. Figure 3c–e shows the simulation models of three cooling schemes, and the geometrical parameters used in the models are shown in Table 1.

Table 2 shows the thermal conductivity of each layer material used in the models. The *TBR* of the heterogeneous interface between GaN and SiC ranges from 4 to 40 m^2^·K·GW^−1^ [35,36]. The *TBR* is assumed to be 10 m^2^·K·GW^−1^ in the models, and the equivalent thermal conductivity of the layer is calculated according to Equation (1).

### 2.2. Mesh Size and Boundary Conditions

**Figure 4:**
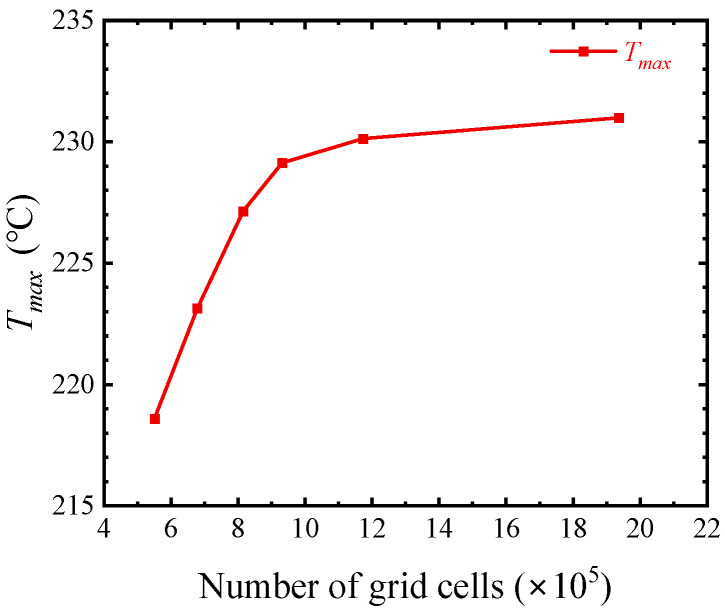
Relationship between the number of grid cells and maximum temperature.

The size of the grid division affects the accuracy and efficiency of numerical calculation. Taking R-cool as an example, the relationship between the number of grid cells and the maximum temperature is shown in Figure 4. As the number of grid cells increases to about 1.2 million, the temperature remains basically unchanged, at which time the free tetrahedral mesh sizes of the key regions, such as the heat source layer, buffer layer, and interface layer are 0.1 μm, 2 μm, and 3 μm, respectively.

The simulation was performed using a steady state type of analysis with a power density of *Q* = 100 MW/cm^3^ loaded on 22 rectangular heat sources with dimensions of 0.5 μm × 150 μm × 0.1 μm. The convective heat transfer coefficient *h_conv_* = 500 kW/m^2^·K was applied at the bottom of the microchannel substrate. The ambient temperature was set to 25 °C, and all other surfaces were assumed to be adiabatic.

### 2.3. Data Processing

Thermal resistance is an important parameter for evaluating thermal characteristics. The thermal resistance *R_i_*_-*c*_ from each layer to the coolant is calculated as follows,

(2) $$R_{i-c}=\frac{\Delta T}{q}=\frac{T_{max}-T_{0}}{q}$$

where *i* is each layer in the heat transfer path; Δ*T* is the temperature difference in reaching thermal equilibrium; *q* is the applied heat flux; *T_max_* is the maximum temperature of *i* layer; and *T*_0_ is the ambient temperature. When *i* is the heat source layer, *R_i_*_-*c*_ is the total thermal resistance in the heat transfer path.

The heat flux *q* is calculated as follows,

(3) $$q=Q\times H_{g}$$

where *Q* is the applied heat source power density and *H_g_* is the heat source thickness.

The thermal resistance *R_i_* of each layer of material in the heat transfer path is calculated as follows,

(4) $$R_{i}=\frac{T_{max1}-T_{max2}}{q}$$

where *T_max_*_1_, *T_max_*_2_ are the maximum temperatures of, respectively, the top and bottom surfaces of each layer of material extracted after reaching thermal equilibrium and *q* is the heat flux at the surface of the applied heat sources.

## 3. Results and Discussion

### 3.1. Cooling Scheme

**Figure 5:**
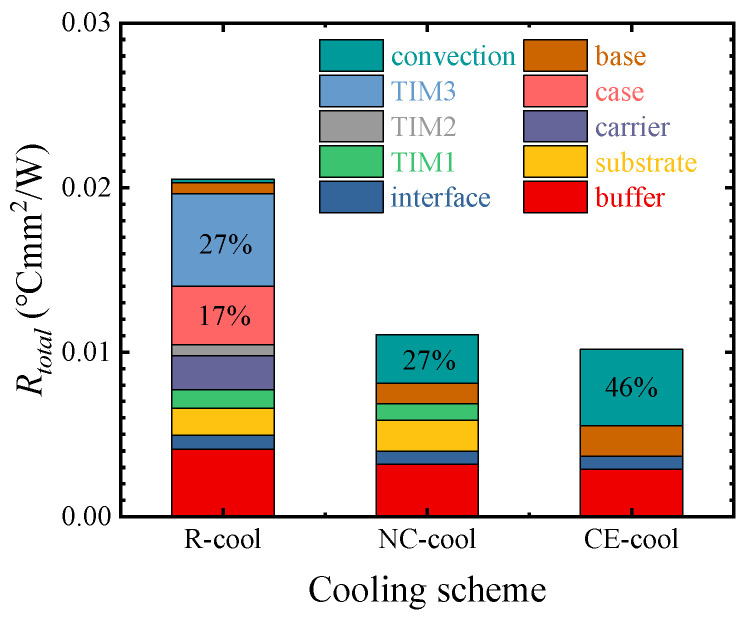
Thermal resistances of three cooling schemes and the ratio of thermal resistance for each material layer.

Figure 5 shows the thermal resistance of three cooling schemes and the ratio of thermal resistance of each material layer. The thermal resistances of R-cool, NC-cool, and CE-cool are, respectively, 0.0205 °Cmm^2^/W, 0.0111 °Cmm^2^/W, and 0.0102 °Cmm^2^/W. The results show that compared with R-cool, NC-cool and CE-cool exhibit significantly lower thermal resistance. It is generally believed that the fewer the number of material layers on the heat transfer path, the smaller the total thermal resistance. However, the thermal resistance of NC-cool is essentially close to that of CE-cool, which is slightly higher by 8.1%, despite the addition of TIM1. The thermal resistance of each layer of materials was calculated using Equation (4). As shown in Figure 5, the ratio of *R_case_* and *R_TIM_*_3_ to the total thermal resistance of R-cool is 44%. The *R_conv_* of NC-cool and CE-cool, respectively, account for 27% and 46% of the total thermal resistance. Therefore, it is crucial to decrease the conduction thermal resistance of the case and TIM3 in R-cool and to decrease the convection thermal resistance in NC-cool and CE-cool.

**Figure 6:**
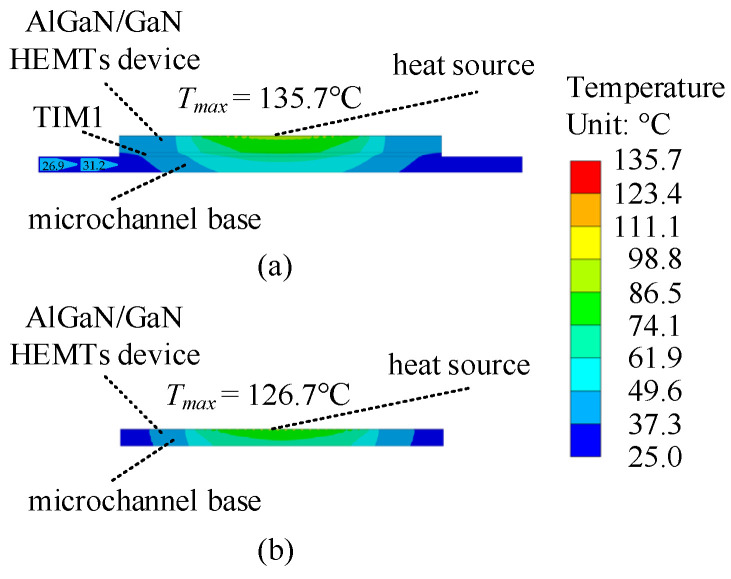
Vertical profile temperature distributions: (**a**) NC-cool and (**b**) CE-cool.

It is shown in Figure 5 that although NC-cool increases the thermal resistance of TIM1 and the substrate layer, the convective thermal resistance is significantly decreased. The vertical profile temperature distributions of NC-cool and CE-cool were extracted and shown in Figure 6. It shows that compared with CE-cool, the transverse heat diffusion distance of the device is longer in NC-cool, which results in a larger effective convective heat transfer area; therefore, the convective thermal resistance is lower.

**Figure 7:**
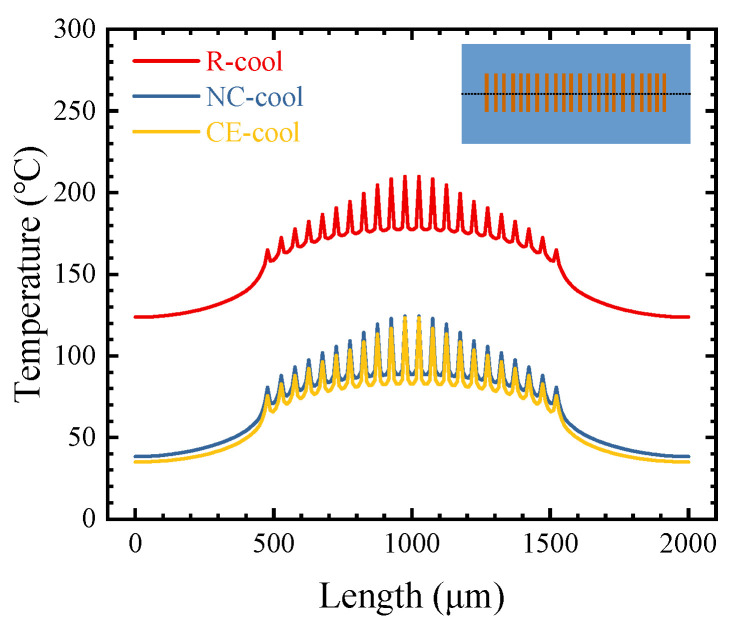
Temperature distributions on the surface of the buffer layer for the three cooling schemes.

Figure 7 shows the temperature distributions on the surface of the buffer layer for the three cooling schemes. The peak temperatures of the center heat source are all significantly higher than those of the edge heat source. The temperature difference between the center heat source and the edge heat source for R-cool, NC-cool, and CE-cool is, respectively, 44.9 °C, 43.7 °C, and 47.7 °C, which are almost the same for the three cooling schemes.

### 3.2. Heat Source Pitch

**Figure 8:**
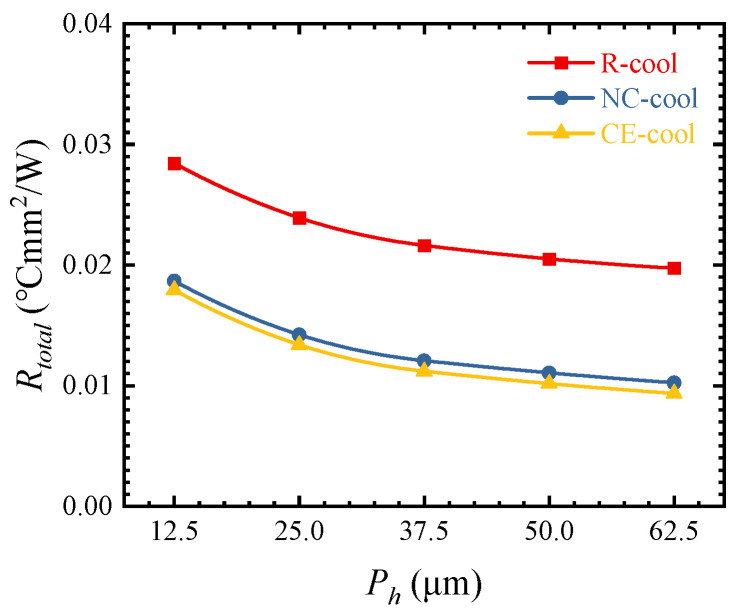
Influences of different *P_h_* values on thermal resistance.

Figure 8 shows the influences of different *P_h_* values on the thermal resistance. With the increase in *P_h_* from 12.5 to 62.5 μm, the thermal resistance of R-cool, NC-cool, and CE-cool decreases by 30.59%, 45.18%, and 47.81%, respectively. With the increase in *P_h_*, the decrease in thermal resistance becomes smaller gradually.

**Figure 9:**
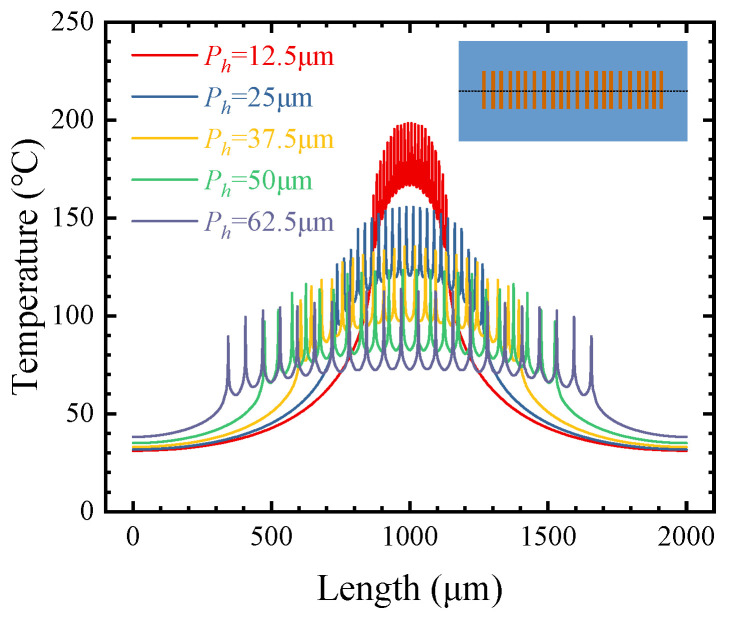
The temperature distributions of different *P_h_* on the surface of the buffer layer in the CE-cool scheme.

The temperature distributions of different *P_h_* values on the surface of the buffer layer were extracted in the CE-cool scheme, as shown in Figure 9. With the increase in *P_h_* from 12.5 to 62.5 μm, the peak temperature of the center heat source decreases from 198.3 to 113.7 °C, and the temperature difference between the most marginal heat source and the center heat source decreases from 37.0 to 24.2 °C. This is because, in the process of heat transferring from a heat source with a small area to a microchannel base with a large area, the transverse diffusion of heat from multiple heat sources will cause the intersection of heat diffusion paths between the heat sources and the superposition of temperatures. By increasing *P_h_*, the temperature superposition decreases and the peak temperature decreases. Therefore, it is necessary to choose a suitable *P_h_* for thermal optimization design.

### 3.3. Substrate and the Convective Heat Transfer Coefficient

**Figure 10:**
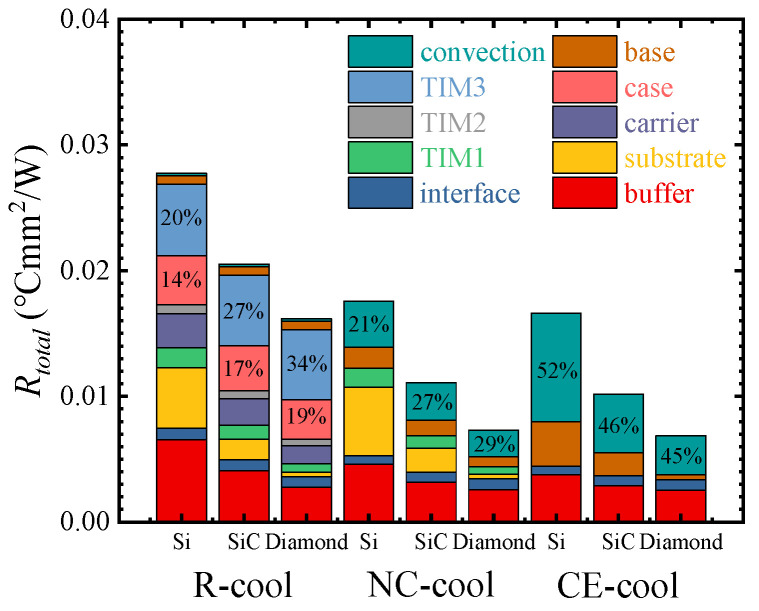
Thermal resistances under different substrates and the ratio of thermal resistance of each material layer in three cooling schemes.

The thermal characteristics of AlGaN/GaN HEMTs with Si, 4H-SiC, and Diamond as substrates were compared. As shown in Figure 10, when the substrate is SiC and Diamond, compared with the Si substrate, the thermal resistance of R-cool decreases by 26.04% and 41.68%, that of NC-cool by 36.97% and 58.36%, and that of CE-cool by 36.62% and 57.23%, respectively. The substrate has a significant influence on the thermal resistance, and the absolute values of the thermal resistance decrease are almost the same. This is because the heat generation by AlGaN/GaN HEMTs devices is characterized by a hotspot heat source with extremely high heat flux and very small area, and a substrate with high thermal conductivity can diffuse the heat gathered in the heat source area faster, and the diffusion thermal resistance is significantly decreased.

In R-cool, *R_case_* and *R_TIM_*_3_ in the Diamond substrate account for a higher proportion of its total thermal resistance compared with the SiC substrate. In NC-cool and CE-cool, the ratio of *R_conv_* in the total thermal resistance shows little change, and it is still the main part of the total thermal resistance. Therefore, decreasing the convective thermal resistance is still the key to enhancing the cooling performance of NC-cool and CE-cool.

**Figure 11:**
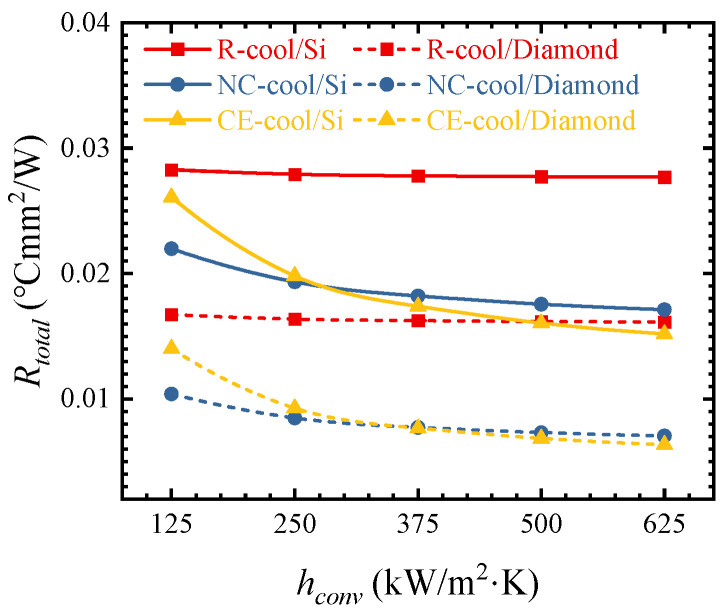
Influences of *h_conv_* on thermal resistance with different substrates and cooling schemes.

Figure 11 shows the influences of different *h_conv_* values on the thermal resistance. With the increase in *h_conv_* from 125 to 625 kW/m^2^·K, the thermal resistance of AlGaN/GaN HEMTs on the Si and Diamond substrates decreases by 22.11% and 32.32% in the NC-cool scheme, while the thermal resistance decreases by 41.79% and 54.68% in the CE-cool scheme, respectively. It can be seen that the convective thermal resistance has a significant influence on the cooling performance of NC-cool and CE-cool, and the influence is more significant when the substrate is Diamond. As can be seen from Figure 10, in the R-cool scheme, the proportion of the convective thermal resistance is very small, so the change in the convective heat transfer coefficient should have little influence on the total thermal resistance. As shown in Figure 11, the thermal resistance in the R-cool scheme is almost constant as the convection heat transfer coefficient increases.

### 3.4. Boundary Thermal Resistance

**Figure 12:**
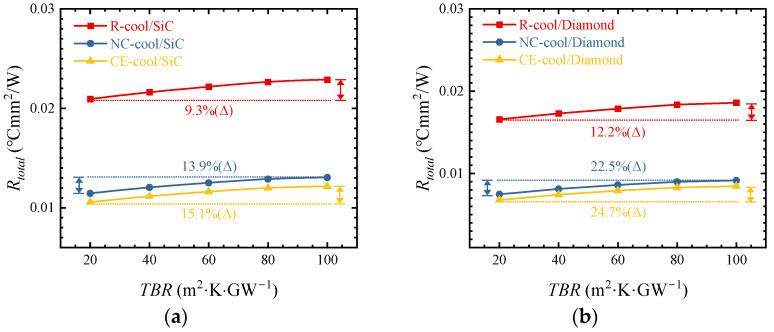
(**a**) Influences of different *TBR* values on thermal resistance with a SiC substrate when *h_conv_* is 500 kW/m^2^·K. (**b**) Influences of different *TBR* values on thermal resistance with a Diamond substrate when *h_conv_* is 625 kW/m^2^·K.

Figure 12a shows the influences of different *TBR* values on thermal resistance when the substrate is SiC and *h_conv_* is 500 kW/m^2^·K. When the *TBR* increases from 20 to 100 m^2^·K·GW^−1^, the thermal resistance increases by 9.3%, 13.9%, and 15.5% in R-cool, NC-cool, and CE-cool. As shown in Figure 12b, when the substrate is Diamond and *h_conv_* increases to 625 kW/m^2^·K, the thermal resistance increases by 12.2%, 22.5%, and 24.7% in R-cool, NC-cool, and CE-cool. In comparison, it can be seen that the higher the *λ_sub_* is and the larger the *h_conv_* is, the larger the increase in thermal resistance with the increase in *TBR*. In the NC-cool and CE-cool schemes, decreasing *TBR* has a more significant influence on the decrease in thermal resistance in the case of high *λ_sub_* and large *h_conv_*.

### 3.5. Height of the Microchannel Base

**Figure 13:**
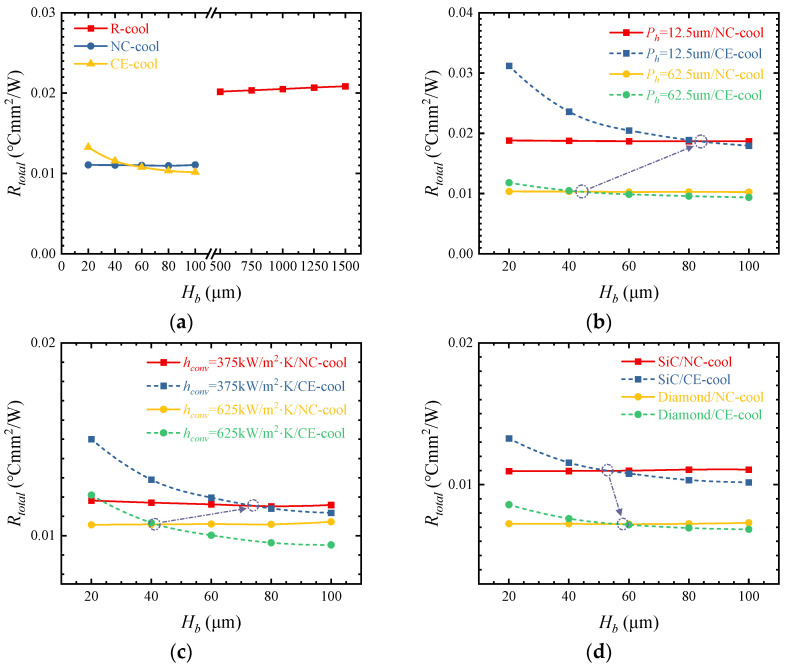
(**a**) Influences of different *H_b_* on thermal resistance. (**b**) Influence of *P_h_* on the thermal resistance intersection. (**c**) Influence of *h_conv_* on the thermal resistance intersection. (**d**) Influence of the substrate material on the thermal resistance intersection.

Figure 13a shows the influences of different *H_b_* values on thermal resistance. As *H_b_*_-*R*_ increases from 500 to 1500 μm, the thermal resistance of R-cool increases by 3.38%. With the increase in *H_b_*_-*N*_ from 20 to 100 μm, the thermal resistance of NC-cool increases by 0.99%. However, when *H_b_*_-*C*_ increases from 20 to 100 μm, the thermal resistance of CE-cool decreases by 23.31%. The results show that increasing the *H_b_*_-*C*_ of CE-cool can achieve a lower thermal resistance. This is because with the increase in *H_b_*_-*C*_, the transverse diffusion of heat transfer from the heat sources to the convective heat transfer interface increases, the convective heat transfer area increases, and the convective thermal resistance significantly decreases with the same convective heat transfer coefficient. As can be seen from Figure 5, convective thermal resistance is the main contributor to the total thermal resistance in CE-cool, and the decrease in convective thermal resistance leads to a significant decrease in the total thermal resistance.

There is an intersection between the thermal resistance curves of NC-cool and CE-cool for different *H_b_*. When the thickness is less than the value at the intersection, NC-cool can achieve better cooling performance, and when the thickness is greater than the value at the intersection, CE-cool can realize lower thermal resistance. However, in CE-cool, a thicker device substrate is required to increase *H_b_* and to fabricate high aspect ratio microchannels to enhance the convective heat transfer coefficient. As shown in Figure 13b–d, when *P_h_* is smaller, *h_conv_* is smaller, or the λ*_sub_* is higher, and the thickness of the intersection of the thermal resistance curves is thicker; therefore, a thicker chip substrate is required to achieve the optimal cooling performance of CE-cool.

## 4. Conclusions

In this paper, the thermal characteristics of the R-cool, NC-cool, and CE-cool schemes for AlGaN/GaN HEMT cooling are compared, and the influences of different geometric parameters and working conditions on thermal resistance are analyzed. The conclusions are as follows:

(1) Compared with R-cool, NC-cool and CE-cool exhibit significantly lower thermal resistance, and the thermal resistance of NC-cool and CE-cool are almost the same. The decrease in *H_b_* significantly increases the thermal resistance of CE-cool, and there exists a critical thickness. When the thickness is less than the critical value, NC-cool can obtain superior cooling performance than CE-cool. The critical thickness decreases with increasing *P_h_* and *h_conv_* or decreasing λ*_sub_*.
(2) Increasing *P_h_* or *λ_sub_* significantly improves the thermal resistance of the three cooling schemes. When *P_h_* increases from 12.5 to 62.5 μm, the thermal resistance of R-cool, NC-cool, and CE-cool decreases by 30.59%, 45.18%, and 47.81%, respectively. When the substrate is Diamond, compared with the Si substrate, the thermal resistance for R-cool, NC-cool, and CE-cool decreases by 41.68%, 58.36%, and 57.23%, respectively.
(3) *h_conv_* has little influence on the thermal resistance of R-cool, whereas it has a significant influence on the thermal resistance of NC-cool and CE-cool. When *h_conv_* increases from 125 to 625 kW/m^2^ K, the thermal resistance of AlGaN/HEMTs on the Diamond substrate decreases by 32.32% and 54.68% in the NC-cool and CE-cool schemes, respectively.
(4) The influence of *TBR* on thermal resistance increases significantly at higher *λ_sub_* and larger *h_conv_*. Under the Diamond substrate with an *h_conv_* of 625 kW/m^2^ K, when *TBR* increases from 20 to 100 m^2^·K·GW^−1^, the thermal resistances of NC-cool and CE-cool increase by 22.5% and 24.7% respectively, while the thermal resistance of R-cool increases by only 12.2%.

## Figures and Tables

**Table 1:** Geometrical parameters used in the models.

Parameter	Definition	Value (μm)
*L_d_*, *W_d_*	Length and width of device	2000, 700
*L_h_*, *W_h_*, *H_h_*	Length, width, and height of heat source	0.5, 150, 0.1
*P_h_*	Heat source pitch	50
*H_buf_*, *H_inter_*, *H_sub_*, *H_TIM_*_1_, *H_car_*, *H_TIM_*_2_, *H_case_*, *H_TIM_*_3_	Height of buffer, interface, substrate, TIM1, carrier, TIM2, case, and TIM3	2, 3, 100, 25, 300, 25, 1000, 50
*L_b_*_-*R*_, *W_b_*_-*R*_, *H_b_*_-*R*_	Length, width, and height of the microchannel base (R-cool)	6000, 3000, 1000
*L_b_*_-*N*_, *W_b_*_-*N*_, *H_b_*_-*N*_	Length, width, and height of the microchannel base (NC-cool)	3000, 1500, 100
*L_b_*_-*C*_, *W_b_*_-*C*_, *H_b_*_-*C*_	Length, width, and height of the microchannel base (CE-cool)	2000, 700, 100

**Table 2:** Thermal conductivity of materials used in the models.

Layer	Meterial	Thermal Conductivity (W/m·K)
heat source, buffer	GaN	150
interface	AlN	X, Y, Z: 0, 0, 300
substrate, carrier, case	4H-SiC, Mo80Cu20, Al50Si50	420, 170, 140
TIM1, TIM2, TIM3	Au80Sn20, Sn63Pb37, silicon grease	57, 51, 1
microchannel base of R-cool, NC-cool, and CE-cool	6061Al, Si, 4H-SiC	155, 148, 420

## Data Availability

Data is contained within the article.
